# The knock‐down of the expression of *MdMLO19* reduces susceptibility to powdery mildew (*Podosphaera leucotricha*) in apple (*Malus domestica*)

**DOI:** 10.1111/pbi.12562

**Published:** 2016-05-11

**Authors:** Stefano Pessina, Dario Angeli, Stefan Martens, Richard G.F. Visser, Yuling Bai, Francesco Salamini, Riccardo Velasco, Henk J. Schouten, Mickael Malnoy

**Affiliations:** ^1^ Research and Innovation Center Fondazione Edmund Mach San Michele all'Adige Italy; ^2^ Wageningen UR Plant Breeding Wageningen University and Research Centre Wageningen The Netherlands

**Keywords:** *MLO*, *Malus domestica*, RNA interference, powdery mildew, *Arabidopsis thaliana*

## Abstract

Varieties resistant to powdery mildew (PM; caused by *Podosphaera leucotricha)* are a major component of sustainable apple production. Resistance can be achieved by knocking‐out susceptibility S‐genes to be singled out among members of the *MLO* (*Mildew Locus O*) gene family. Candidates are *MLO* S‐genes of phylogenetic clade V up‐regulated upon PM inoculation, such as *MdMLO11* and *19* (clade V) and *MdMLO18* (clade VII). We report the knock‐down through RNA interference of *MdMLO11* and *19*, as well as the complementation of resistance with *MdMLO18* in the *Arabidopsis thaliana* triple *mlo* mutant *Atmlo2/6/12*. The knock‐down of *MdMLO19* reduced PM disease severity by 75%, whereas the knock‐down of *MdMLO11,* alone or in combination with *MdMLO19,* did not result in any reduction or additional reduction of susceptibility compared with *MdMLO19* alone. The test in *A. thaliana* excluded a role for *MdMLO18* in PM susceptibility. Cell wall appositions (papillae) were present in both PM‐resistant and PM‐susceptible plants, but were larger in resistant lines. No obvious negative phenotype was observed in plants with *mlo* genes knocked down. Apparently, *MdMLO19* plays the pivotal role in apple PM susceptibility and its knock‐down induces a very significant level of resistance.

## Introduction

Powdery mildew (PM), caused by the obligate biotrophic fungus *Podosphaera leucotricha,* is a major disease of *Malus domestica* present in all major apple‐growing areas of the world (Turechek *et al*., 2004). Leaves are the most susceptible organs, particularly in the first days after opening. Powdery white lesions present on the upper leaf side eventually turn brown, whereas infections on the underside result in chlorotic patches. Infected leaves tend to crinkle, curl and drop prematurely (Turechek *et al*., 2004). Blossom infections are less common but important because infected fruits are small and stunted if not dropping. *P. leucotricha* survives the winter as mycelium in vegetative tissues or in infected flower buds (Turechek *et al*., 2004). The primary infection starts when infected buds break dormancy: the fungus resumes growth and colonizes developing shoots. Primary infections of flower buds cause severe yield losses. Spores growing on infected shoots spread nearby and initiate secondary infections (Turechek *et al*., 2004).

Yield losses caused by PM can be limited with frequent applications of fungicides. However, fungicides, besides their significant cost for the growers, affect the environment negatively (Wightwick *et al*., 2010). Moreover, agrochemical treatments select fungicide‐resistant strains of the pathogen, as known for *Erysiphe necator*, the PM causing agent of grapevine (Dufour *et al*., 2011), and *Venturia inaequalis,* the agent of apple scab (Pfeiffer *et al*., 2015). Therefore, the development of PM‐resistant varieties is a valuable option to improve economic and environmental sustainability of apple cultivation.

Apple germplasm, including domesticated and wild *Malus* species, is rich in dominant resistance genes (R‐genes). About 868 R‐genes have been identified in the apple genome, which are effective against a large number of pathogenic organisms (Perazzolli *et al*., 2014). They encode proteins that recognize pathogen effectors and activate the defence response (Dodds and Rathjen, 2010; Pavan *et al*., 2010), manifested as localized hypersensitive response at the site of infection (Bari and Jones, 2009). Two PM R‐genes, *Pl‐1* from *Malus robusta* and *Pl‐2* from *Malus zumi*, have been used since 1970s, in a variety of breeding programmes (Bus *et al*., 2010), later together with *Pl‐m*,* Pl‐w* and *Pl‐d* (James *et al*., 2004; Lespinasse, 1983). Unfortunately, the durability of R‐genes is limited due to new pathogen strains able to overcome the resistance (Parlevliet, 1993), as noted for apple *Pl‐2* and *Pl‐m* (Caffier and Laurens, 2005). Considering how time‐consuming breeding of woody species is, a more durable source of PM resistance is a necessity. This source can be based on mutations in plant susceptibility genes (S‐genes), which are defined as plant genes that are required by pathogens to promote diseases. Some S‐genes encode negative regulators of the plant immunity system, whose impairment prevents the suppression of plant defence and leads to resistance (Pavan *et al*., 2010). However, knocking‐out S‐genes may induce pleiotropic phenotypes in the plant, which may result in negative effects (Pavan *et al*., 2011; Van Schie and Takken, 2014).

The barley *MLO* gene is an example of an S‐gene causing PM susceptibility. The *mlo* recessive resistance caused by the knockout of a dominant *MLO* allele was discovered in barley in 1942 (Jørgensen, 1992) and was for a long time considered a unique form of resistance. Further studies revealed that *MLO* genes are largely conserved across the plant kingdom, as proven in *Arabidopsis thaliana* (Consonni *et al*., 2006), pea (Pavan *et al*., 2011), tomato (Bai *et al*., 2008), wheat (Wang *et al*., 2014), pepper (Zheng *et al*., 2013) and grapevine (S. Pessina *et al.,* unpublished). Genes of the *MLO* family define seven phylogenetic clades (Acevedo‐Garcia *et al*., 2014; Pessina *et al*., 2014) of which only two include S‐genes: clade IV, with *MLO* S‐genes of monocots (Panstruga, 2005; Reinstädler *et al*., 2010), and clade V, with *MLO* S‐genes of dicots (Bai *et al*., 2008; Consonni *et al*., 2006; Feechan *et al*., 2008; Winterhagen *et al*., 2008). However, not all members of clades IV and V are S‐genes, but nevertheless candidates can be identified during early stages of PM infection because of an increased expression, as documented in tomato (Bai *et al*., 2008), barley (Piffanelli *et al*., 2002), pepper (Zheng *et al*., 2013), grapevine (Feechan *et al*., 2008; Winterhagen *et al*., 2008) and apple (Pessina *et al*., 2014). In the latter species, four *MLO* genes belong to clade V and two of them, *MdMLO11* and *MdMLO19,* are up‐regulated during PM infection, whereas *MdMLO5* and *MdMLO7* are not transcriptionally responsive to the pathogen (Pessina *et al*., 2014). In addition, *MdMLO18*, a gene belonging to clade VII, is also up‐regulated (Pessina *et al*., 2014). To date, there are no reports of *MLO* genes of dicots acting as S‐genes outside clade V; therefore, *MdMLO18* should not be a strong candidate for being an S‐gene.

MLO proteins have seven trans‐membrane domains and are involved in a variety of physiological processes in different tissues (Acevedo‐Garcia *et al*., 2014). The proposed function for MLO S‐proteins is the negative regulation of vesicle‐associated and actin‐dependent defence pathways at the site of attempted PM penetration (Panstruga, 2005). Plant *mlo‐*based resistance is associated with cell wall appositions called papillae that constitute a mechanical barrier for the pathogen. Therefore, *mlo* resistance consists of a prepenetration structural defence system (Aist and Bushnell, 1991; Consonni *et al*., 2006). The formation of the papillae depends on the delivery of material through the actin‐dependent vesicle traffic (Feechan *et al*., 2011; Miklis *et al*., 2007). In *A. thaliana, MLO* genes have other functions: *AtMLO7* is involved in pollen tube reception by the embryo sac (Kessler *et al*., 2010), whereas *AtMLO4* and *AtMLO11* participate in the control of root architecture (Chen *et al*., 2009).

The development of DNA editing tools is rapidly changing plant genetics and biotechnology, thanks to the possibility of inducing mutations in specific genes (Gaj *et al*., 2013; Lozano‐Juste and Cutler, 2014; Puchta and Fauser, 2014). Targeted knockout of *MLO* S‐genes, using DNA editing tools, may provide durable resistance to PM in apple, but, before applying the gene editing approach, evidence of which *MLO* gene(s) cause PM susceptibility in apple is required. This study reports the functional analysis on apple *MLO* genes, *MdMLO*11, *18* and *19* for their roles in susceptibility to PM, by knocking‐down *MdMLO11* and *19* through RNA interference (RNAi) and overexpressing *MdMLO18* in the *Arabidopsis Atmlo2/6/12* mutant.

## Results

### Overexpression of *MdMLO18* in *A. thaliana* triple *mlo* mutant did not increase susceptibility

**Figure 1:**
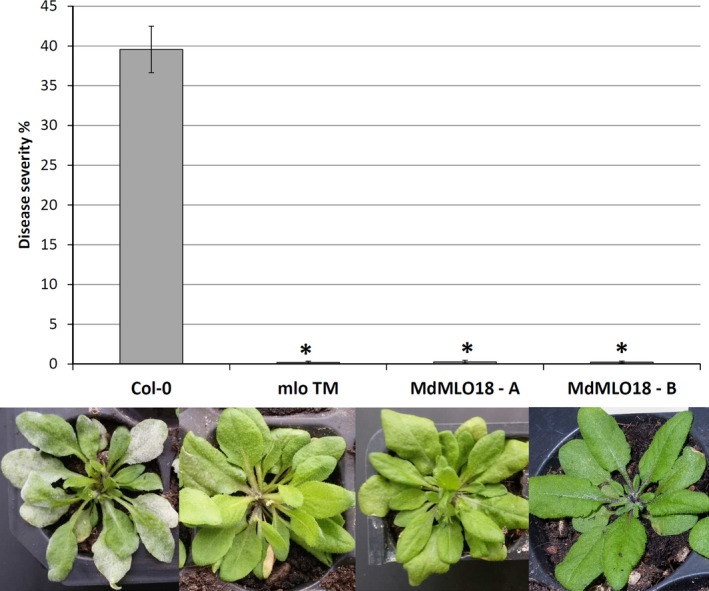
Disease severity recorded after 7 days from the inoculation with *O. neolycopersici* of *A. thaliana* Col‐0, *A. thaliana Atmlo2/6/12* mutant, *A. thaliana Atmlo2/6/12* mutants expressing *MdMLO18* line A and *A. thaliana Atmlo2/6/12* mutants expressing *MdMLO18* line B. Histograms, representing average PM severity, show data of 7 to 24 biological replicates. Error bars show the standard errors of the mean. The asterisks indicate statistically significant differences compared with Gala, according to the Kruskal–Wallis test (*P* = 0.01).

Two PM‐resistant *A. thaliana Atmlo2/6/12* mutants overexpressing *MdMLO18* (lines A and B) were generated via *A. tumefaciens* transformation by floral dipping. Seedlings of *Atmlo2/6/12*, of the two *Atmlo2/6/12‐MdMLO18* lines and of *A. thaliana* Col‐0 were inoculated with *O. neolycopersici*. Seven days after inoculation, no infection was detected on the leaves of neither *Atmlo2/6/12* nor *Atmlo2/6/12‐MdMLO18*, whereas *A. thaliana* Col‐0 was heavily infected (Figures 1 and S1). This result suggests that *MdMLO18* does not have a role in PM susceptibility of apple.

### Development of RNAi apple plantlets

Three RNAi constructs were generated, two aimed at knocking‐down *MdMLO11* (construct A = KD‐*MdMLO11*) and *MdMLO19* (construct B = KD‐*MdMLO19*) individually and the third aimed at the simultaneous knock‐down of *MdMLO11* and *MdMLO19* (construct C = KD‐*MdMLO11+19*). Of the 80 regenerated lines obtained, 48 did carry the RNAi insert as described in Materials and Methods (Table S1). Seven lines were lost due to *in vitro* contamination before it was possible to test them. The other 41 *in vitro* transgenic lines were tested by qPCR to evaluate the level of *MLO* gene expression. Significant knock‐down for *MdMLO11* was detected in three lines, whereas for *MdMLO19* in ten lines (Figure S2). Furthermore, five lines showed increased expression for *MdMLO11* (Figure S2). Sixteen lines were acclimated to glasshouse conditions, with a survival rate above 90%. The only exception was line GT.B‐2 (Gene Transfer B), which was not able to survive to glasshouse conditions. A further gene expression analysis on sample collected in the glasshouse confirmed a significant gene knock‐down in only three of the 15 lines successfully acclimated (Figure S3 and Table S1). The up‐regulation of *MdMLO11* was not confirmed in any transgenic line. In the three lines showing knock‐down for *MdMLO11* and *19*, no off‐target effect was detected for the other two clade V genes of apple (*MdMLO5* and *7*). The three knock‐down lines were named TG11 (transgenic Gala *MdMLO11*), TG19 (transgenic Gala *MdMLO19*) and TG11+19 (transgenic Gala *MdMLO11+19*). Two further lines were acclimated: the control ‘Gala’ and TG0, a line carrying the RNAi construct for *MdMLO19* but not showing significant *MLO* genes knock‐down. TG0, TG11, TG19 and TG11+19 are indicated as transgenic lines, but only TG11, TG19 and TG11+19 as *mlo* lines.

Under glasshouse conditions, the *mlo* lines showed a normal growth compared with ‘Gala’.

### Reduced susceptibility to *P. leucotricha* of RNAi apple plants

**Figure 2:**
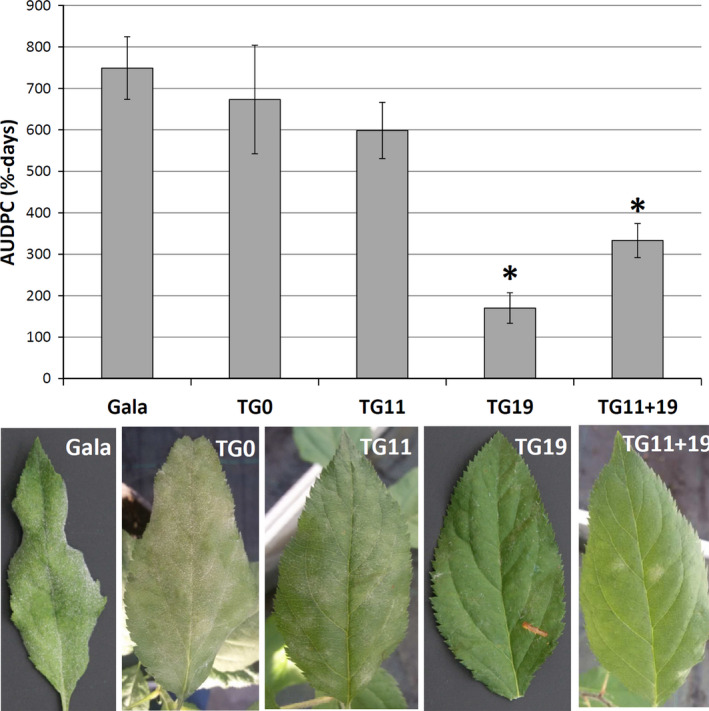
Area under disease progress curve (AUDPC) of four *mlo* lines and of the control ‘Gala’, inoculated with *P. leucotricha*. Average AUDPC was calculated from 15 to 24 biological replicates considered in four experiments. Error bars show the standard errors of the mean. Statistically significant differences in the comparisons with ‘Gala’, according to the Tukey and Games–Howell *post hoc* tests (*P* = 0.05), are indicated with asterisks. Leaves were collected at 21 dpi between the fourth and eighth node and avoiding the new leaves that opened after the inoculation.

**Figure 3:**
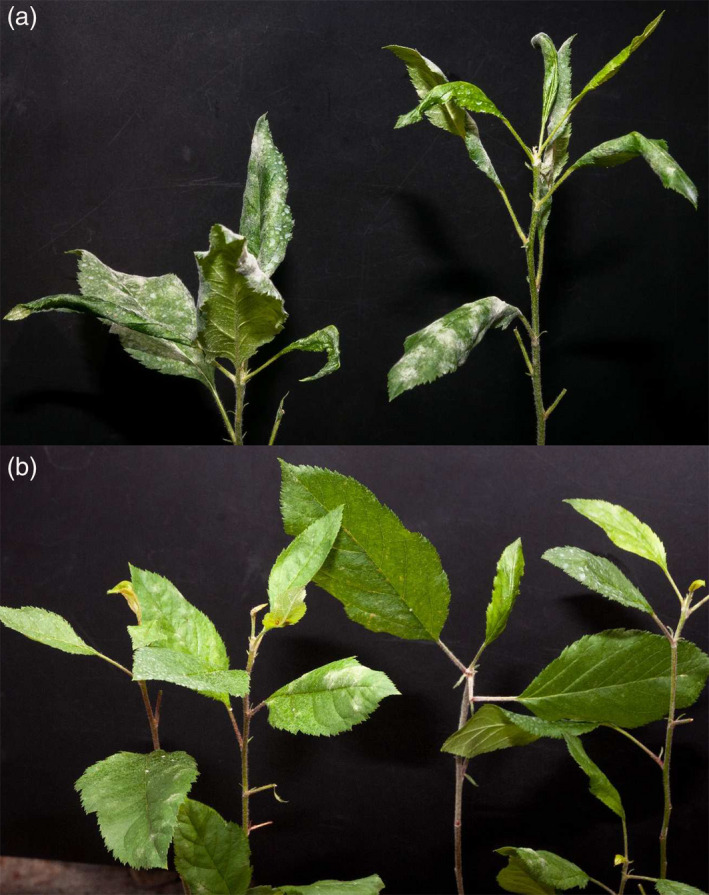
(a) Two ‘Gala’ plants photographed at 21 dpi. Leaves are heavily infected and the majority of them are crinkled, including the young ones. (b) Three TG19 plants photographed at 21 dpi. Leaves are moderately or not infected, with no sign of crinkling.

The four transgenic lines and the control were tested for their susceptibility to PM in four independent experiments. Although some variations in the progress of infection were observed between different seasons, that is the infection was slower in winter, the general effects were of a similar nature and not significant. TG0, the line not manifesting any *MLO* genes knock‐down, showed a level of susceptibility to *P. leucotricha* comparable to that of the control. The same was noted for TG11, whereas TG11+19 and TG19 had an evident reduction of disease severity (Figures 2, 3 and S4). Although leaves of TG11+19 and TG19 plants were partially infected (Figures 2, 3 and S4), the extension of the adaxial leaf area covered in spores was significantly reduced compared with the control (Figures 2, 3 and S4). Furthermore, TG19 and TG11+19 plants were more vigorous and did not show any sign of leaf crinkling and curling (Figure 3). Table 1 summarizes the disease severity reduction.

**Table 1:** PM disease severity reduction (%) in lines transformed with *MLO* RNAi constructs

Line	Silenced genes	Replicates	Disease severity reduction^b^		
			14 dpi	21 dpi	Average
TG0	/	17	24.1	24.8	24.5
TG11+19	*MdMLO11* and *19*	23	60.0^a^	52.6^a^	56.3
TG19	*MdMLO19*	15	72.7^a^	78.1^a^	75.4
TG11	*MdMLO11*	16	38.0	−3.2^c^	17.4

a Statistically significant difference compared to the control, according to the Tukey *post hoc* test (*P *= 0.05).

b Gala was used as control (19 plants) and assumed to have 0% of disease reduction.

c Line TG11 showed a higher level of infection compared with Gala at 21 dpi.

All the transgenic lines had a reduction in the number of conidia present on leaves (Figure S5), but the decrease was statistically significant (*P* < 0.05) only for TG11+19 and TG19. This compares well with the disease severity assessment presented in Figures 2, 3 and S4: compared with ‘Gala’, TG11+19 showed a 63.3% reduction in the number of conidia and TG19 showed 64.8%. A significant (*P* = 0.01) but moderate positive correlation (Pearson's coefficient of 0.525) was found between disease severity at 21 dpi and conidia count at 21 dpi.

**Figure 4:**
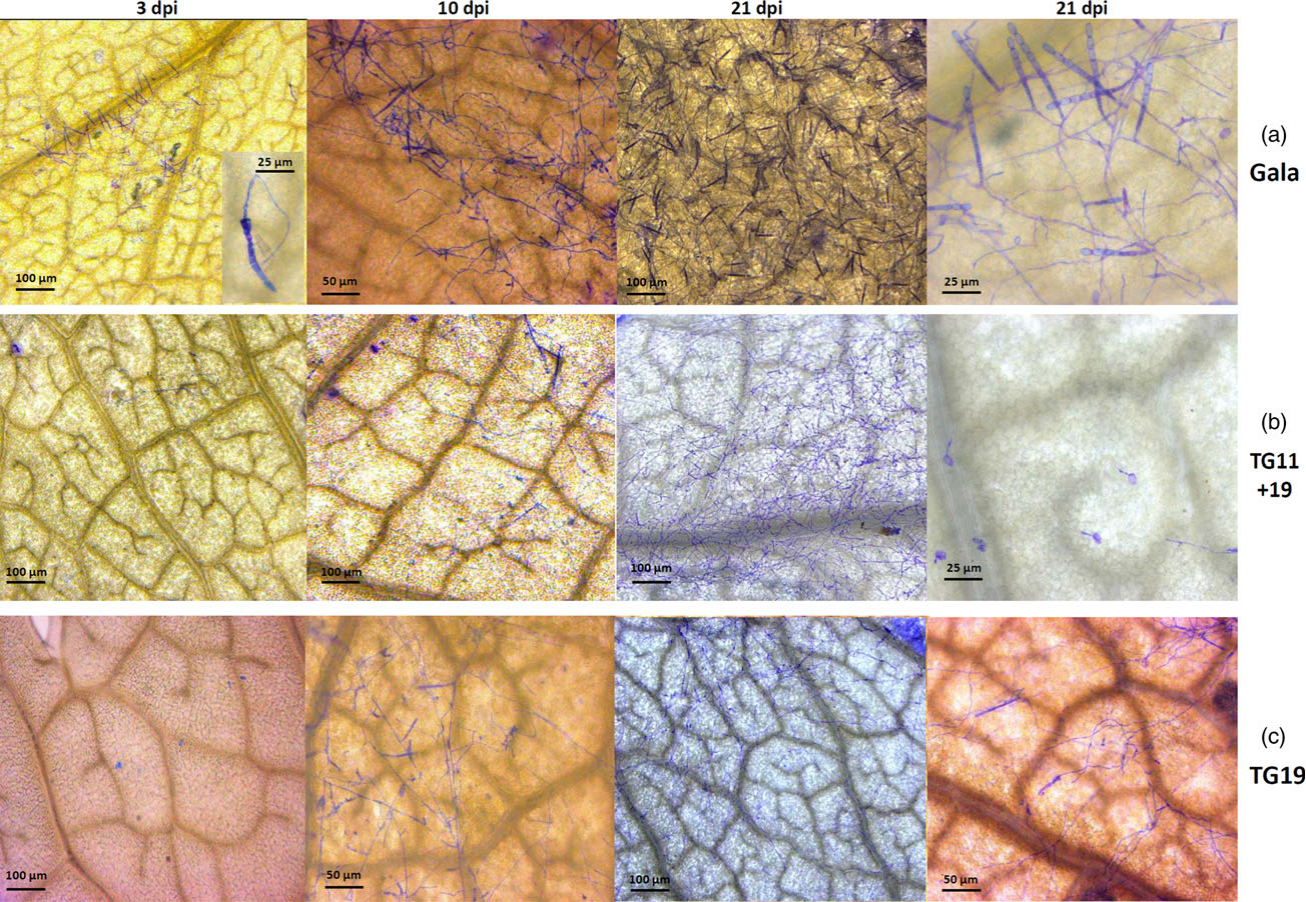
Bright‐field microscopy images of infected leaves of ‘Gala’ (a) and lines TG11+19 (b) and TG19 (c) taken at 3, 10 and 21 dpi. For Gala at 3 dpi, at higher magnification the germination of a *P. leucotricha* spore is shown.

Lines TG11+19 and TG19, together with ‘Gala’, were further analysed by bright‐field microscopy and scanning electron microscopy (SEM), to follow the development of *P. leucotricha* infection. In ‘Gala’, a well‐developed leaf infection was observed already at 3 dpi (Figure 4a), at the time when fungal development was still limited in TG11+19 and TG19 (Figure 4b and c). At 10 dpi, conidiophores were observed on leaves of all lines considered, but they appeared to be denser in ‘Gala’ (Figure 4). At 21 dpi, ‘Gala’ leaves were completely covered by spores and a large number of conidiophores were visible (Figure 4a). The leaf surface of TG11+19 and TG19 was partially colonized by sporulating mycelium, but isolated spores unable to develop were also observed, as well as a smaller number of conidiophores compared with the situation noted for ‘Gala’ (Figure 4b and c). The results of the conidia count are in accordance with these observations (Figure S5). The SEM images showed reduced growth of the mycelium on TG11+19 compared with TG0 and ‘Gala’ (Figure S6).

**Figure 5:**
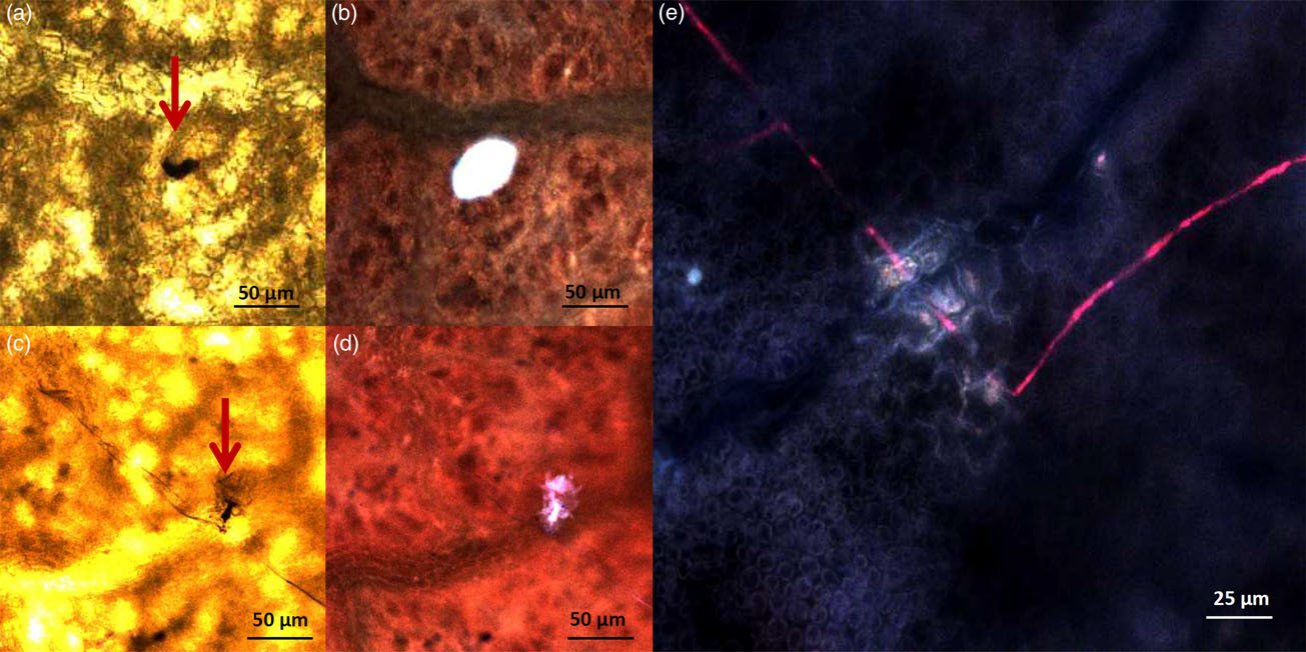
Formation at 3 dpi of papillae in infected leaves of ‘Gala’ (a, b) and in resistant lines TG11+19 (c, d) and TG19 (e). Images on the left were taken with a bright‐field microscope, those on the right with fluorescence microscope. For line TG19, only the image taken with the fluorescent microscope is shown.

The formation of papilla was observed at 3 dpi in all the lines, both resistant and susceptible (Figure 5). The papillae in ‘Gala’ had similar size compared to the ones in TG11+19 (55 and 50 μm, respectively; Figure 5b and d), whereas the papillae in TG19 were bigger (75 μm; Figure 5e). Furthermore, the shape of papillae in ‘Gala’ was more defined (Figure 5c–e).

### Expression of *MLO* genes in *mlo* apple lines

**Figure 6:**
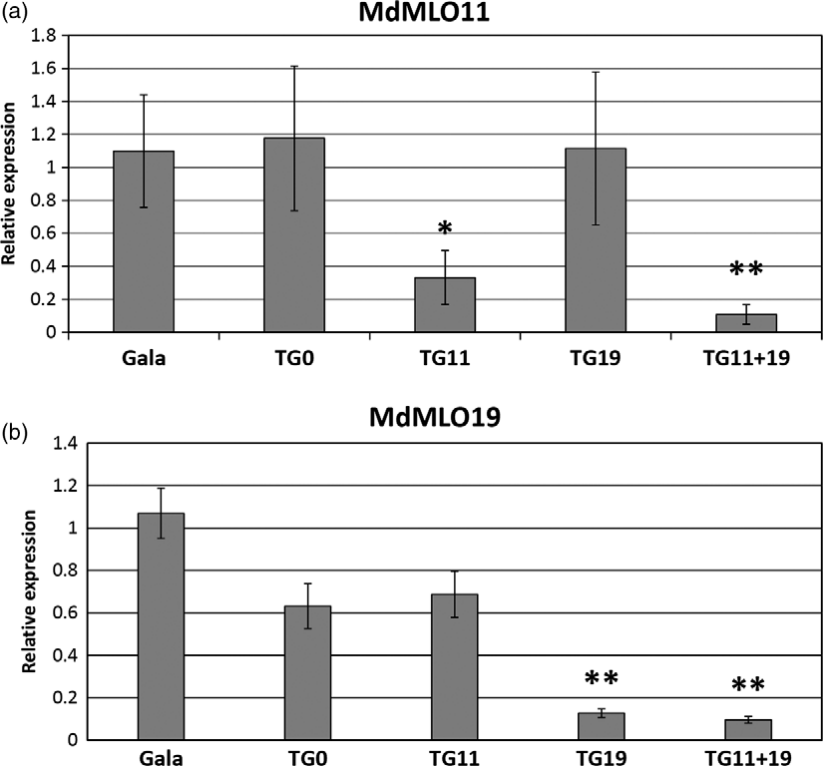
Expression of *MdMLO11* (a) and *MdMLO19* (b) in five lines in the absence of *P. leucotricha* infection. Each bar represents the line average relative expression, evaluated from three to five plants. Error bars show the standard errors of the mean. Asterisks indicate significant differences in the comparison of *mlo* lines with ‘Gala’, based on the Tukey or Games–Howell *post hoc* tests (*P* = 0.05).

Gene expression analysis of *mlo* lines previously selected was repeated in glasshouse‐acclimated plants. *MdMLO11* was significantly less expressed in TG11+19 (*P* = 0.01) and TG11 (*P* = 0.05) (Figure 6a), whereas the expression of *MdMLO19* was reduced in TG11+19 (*P* = 0.01) and TG19 (*P* = 0.01) (Figure 6b). *MdMLO5* and *MdMLO7,* the two other apple members of clade V, were also tested, but no significant reduction was observed in any transgenic line, a finding supporting the absence of off‐target silencing (Figure S7).

Correlation between the expression of *MdMLO19* and AUDPC—a measure of disease severity—was statistically significant (*P* = 0.05), although moderate (Pearson's coefficient = 0.515). On the contrary, no significant correlation was found between AUDPC and the expression of *MdMLO11*.

### Gene expression analysis of *mlo* apple lines TG11+19 and TG19

**Figure 7:**
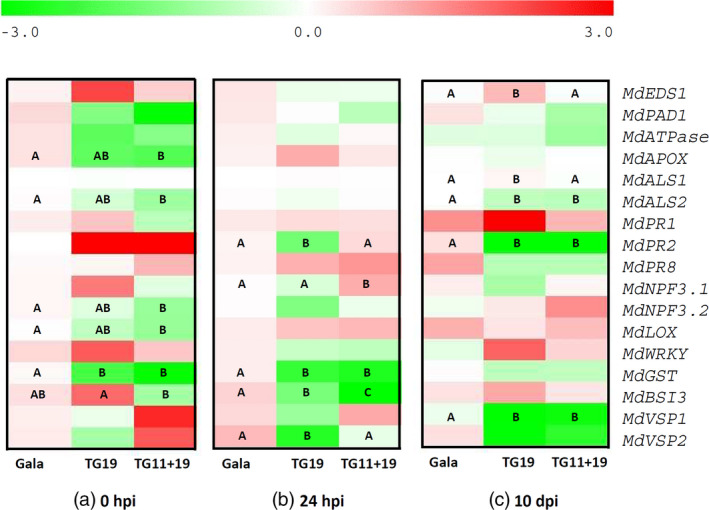
Relative expression in ‘Gala’ and in resistant *mlo* lines TG11+19 and TG19 of 17 plant genes, monitored before PM inoculation (a) and at 24 h (b) and 10 days (c) after the inoculation. The average values of relative expression of ‘Gala’ were used as reference for statistical analyses. The letter code indicates statistically significant differences among time points, according to the Fisher *post hoc* test (*P* = 0.05). Image was prepared with the MultiExperiment Viewer software, with Log2 of relative expression data.

The expression profile of 17 genes related to plant disease resistance was tested at three time points in resistant *mlo* lines TG11+19 and TG19 compared with ‘Gala’ (Figures 7 and S8). These genes were selected because of their role in the interaction with the PM pathogen and in defence in general. In the absence of infection, five genes were down‐regulated in TG11+19 compared with ‘Gala’ and only one in TG19 (Figure 7a). At 24 h postinoculation, the three lines showed only moderate differences: four genes were less expressed in TG19 than in ‘Gala’, whereas in TG11+19 one gene was up‐regulated and two were down‐regulated (Figure 7b). The scenario was slightly different at 10 dpi: three genes were less expressed than in ‘Gala’ and two moderately up‐regulated in TG19 (Figure 7c), whereas three genes were down‐regulated in TG11+19 (Figure 7c).

The effect of *P. leucotricha* inoculation on single lines was different: at 24 hpi, five genes were up‐regulated in ‘Gala’ (Figure S8a), 13 in TG11+19 (Figure S8b) and four in TG19 (Figure S8c). The only gene up‐regulated at 24 hpi in all lines was *MdVSP1* (*vegetative storage protein*) (Figure S8). Of genes up‐regulated at 24 hpi, only few remained induced at 10 dpi: two in ‘Gala’ (Figure S8a) and TG19 (Figure S8c) and three in TG11+19 (Figure S8b).

### Phenolic metabolites composition of *mlo* apple leaves

Of the 135 phenolic secondary metabolites of apple identified by Vrhovsek *et al*. (2012), only 18 were found and quantified in the leaves of ‘Gala’, TG11+19 and TG19 (Table S2). Statistically significant differences between the *mlo* lines TG11+19 and TG19 and ‘Gala’ were noted for chlorogenic acid, rutin (quercetin‐3‐*O*‐rutinoside), kaempferol‐3‐*O*‐rutinoside and isorhamnetin‐3‐*O*‐glucoside (Figure S9). Chlorogenic acid and rutin were lower in both *mlo* lines, but the difference was significant only for chlorogenic acid in TG11+19 (*P* = 0.01) and for rutin in TG19 (*P* = 0.01) (Figure S9a and b). Kaempferol‐3‐*O*‐rutinoside was higher in TG19 (*P* = 0.05) (Figure S9c), as for isorhamnetin‐3‐*O*‐glucoside in both *mlo* lines (*P* = 0.01) (Figure S9d). Compounds derived from the same precursor were also considered together: quercetins (quercetin‐3‐*O*‐rhamnoside + quercetin‐3‐*O*‐glucoside + quercetin‐3‐*O*‐galactoside + rutin) and kaempferols (kaempferol + kaempferol‐3‐*O*‐glucoside + kaempferol‐3‐*O*‐rutinoside) did not show any significant change (Figure S9e and f), whereas isorhamnetins (isorhamnetin + isorhamnetin‐3‐*O*‐glucoside + isorhamnetin‐3‐*O*‐rutinoside) were higher in TG19 (Figure S9g). Quercetins, kaempferols and isorhamnetins, flavonoids of the flavonol subgroup, considered together did not reveal significant differences between ‘Gala’ and *mlo*‐resistant lines (Figure S9h).

## Discussion

Natural and artificial loss‐of‐function mutations of *MLO* S*‐*genes reduce susceptibility to PM pathogens, as described in barley (Büschges *et al*., 1997), *A. thaliana* (Consonni *et al*., 2006), pea (Pavan *et al*., 2011), tomato (Bai *et al*., 2008) and pepper (Zheng *et al*., 2013). In dicots, all PM susceptibility genes belong to clade V (Bai *et al*., 2008; Consonni *et al*., 2006; Feechan *et al*., 2008; Winterhagen *et al*., 2008). In a previous contribution, we identified three *MLO* genes of *M. domestica* up‐regulated during early stages of PM infection (Pessina *et al*., 2014). Two of them, *MdMLO11* and *MdMLO19*, belong to dicot clade V and *MdMLO18* to clade VII. Because *MLO* genes outside clade V acting as S‐genes are not known, only *MdMLO11* and *MdMLO19* were considered reasonable candidates to be knocked down in apple*,* whereas *MdMLO18* was tested with the quicker complementation test of the *A. thaliana* mutan*t Atmlo2/6/12,* which is completely resistant to different nonadapted PM species (Consonni *et al*., 2006), including *O. neolycopersici*, the causal agent of tomato PM (Zheng *et al*., 2013). Complementation is based on the ability of PM pathogens to start a successful infection harnessing *MLO* genes similar, but not identical to the resident ones (Acevedo‐Garcia *et al*., 2014). In case the resistant *Atmlo2/6/12* mutant expressing the foreign *MLO* gene becomes susceptible to PM, it is a first indication that the introduced *MLO* can functionally substitute the native *MLO* S‐genes of *A. thaliana*. *MdMLO18* failed to complement, in accordance with the robust evidence that only clade V genes act as S‐genes in dicots (Acevedo‐Garcia *et al*., 2014; Bai *et al*., 2008; Consonni *et al*., 2006; Humphry *et al*., 2011; Pavan *et al*., 2011; Zheng *et al*., 2013). Therefore, we did not perform RNAi in apple for *MdMLO18*.

*MdMLO11* and *MdMLO19* were knocked down to assess their role in supporting apple susceptibility to PM. RNAi was adopted to reduce the expression of the two *MLO* genes, and in spite of the high number of transgenic ‘Gala’ lines generated (48), a significant reduction in the expression of the target genes was detected in only three of them. In part, this was expected because short RNAi fragments of less than 150 bp, like those used in our experiments, are known for their limited knock‐down efficiency. The use of long fragments of 400 bp or more can result in a high knock‐down efficiency, as reported by Glissen *et al*. (2005) and Flachowsky *et al*. (2012). However, both these studies were carried out on apple cultivars different from ‘Gala’ using different plasmids from those used in this work. Therefore, these two factors, particularly the apple cultivar, might also have contributed to the different knock‐down efficiency. On the other hand, short fragments have the advantage of being more specific, thus avoiding off‐target silencing on other clade V *MLO* genes, as detected in our experiments. As no off‐target knock‐down was detected for *MdMLO5* and *7*, the genes sharing the highest sequence identity with *MdMLO11* and *19* (Pessina *et al*., 2014), it is unlikely that the constructs used in this study caused off‐target knock‐down on other genes.

In some species, knockout or knock‐down of *MLO* genes causes pleiotropic phenotypes, such as formation of necrotic spots on leaves and reduced grain yield in barley (Jørgensen, 1992), slow growth in *A. thaliana* (Consonni *et al*., 2006) and reduced plant size in pepper (Zheng *et al*., 2013). Such or other unexpected pleiotropic phenotypes were not observed under the glasshouse conditions specified in Materials and Methods.

The inoculation of apple transgenic lines with *P. leucotricha* in the glasshouse resulted in a reduction of disease severity in lines TG11+19 and TG19 compared with ‘Gala’. This reduction was statistically significant (*P* = 0.05). To our knowledge, only one other study aimed at achieving PM resistance in apple through gene transfer technologies, specifically overexpressing an R‐gene in ‘Fuji’ (Chen *et al*., 2012). This approach is different from ours and hard to compare. Furthermore, Chen *et al*. (2012) inoculated detached leaves and did not carry out any statistical analysis that would facilitate a direct comparison. The knock‐down of *MdMLO19* in both resistant lines led to the conclusion that this was the gene responsible for the reduction of PM susceptibility. The knock‐down of *MdMLO11* did not result in a significant reduction of susceptibility, and even its knock‐down in combination with *MdMLO19* resulted in any additional reduction of susceptibility. The conclusion is that of the two clade V genes induced by PM in apple, only *MdMLO19* is a functional S‐gene. Also *MdMLO18*, the clade VII gene inducible by *P. leucotricha* inoculation, should not be considered a PM S‐gene. The observation that the mutation of a single *MLO* gene suffices to confer PM resistance in apple is in agreement with previous studies on barley (Jørgensen, 1992), pea (Humphry *et al*., 2011) and tomato (Bai *et al*., 2008). In contrast, in *Arabidopsis*, full PM resistance requires the simultaneous loss of function of three M*LO* homologues (*AtMlo2, AtMlo6* and *AtMlo12*). Line TG0 was considered with the purpose of assessing the effect on susceptibility to PM of the insertion of a ‘target ineffective’ RNAi construct. TG0 was obtained from a transfer that aimed to knock down *MdMLO19*. In this line, no significant decrease in the expression of *MdMLO19* was recorded, as well as no significant reduction of PM susceptibility. It is concluded that the insertion of an ‘ineffective’ RNAi did not have functional relevance.

The precise mechanism through which the loss of function of *MLO* S‐genes reduces susceptibility to PM pathogens is not completely clear yet. However, *mlo* resistance is known to be linked to secretory vesicle trafficking (Feechan *et al*., 2011; Miklis *et al*., 2007) and to the formation of cell wall appositions called papillae (Consonni *et al*., 2006). Papillae consist in a callose matrix enriched in proteins and autofluorogenic phenolics (Vanacker *et al*., 2000) whose formation depends on actin‐dependent endomembrane transport (Hückelhoven, 2014). Lines ‘Gala’, TG11+19 and TG19 were characterized by the presence of papillae at 3 dpi, but shape and dimensions were different in resistant and susceptible lines. Rapid papilla formation (Lyngkjᴂr *et al*., 2000), increased papilla size at attempted penetration sites (Stolzenburg *et al*., 1984) and different biochemical composition (Chowdhury *et al*., 2014), may explain the noted differences between effective and noneffective papillae. In TG19, the size of papillae was larger than in the control, supporting the hypothesis that larger dimensions increase the efficacy of the papilla. Chowdhury *et al*. (2014) have shown that the difference between effective and noneffective papillae lies in the higher concentration of callose, cellulose and arabinoxylan of the effective ones. The observed differences between papillae of resistant and susceptible lines could reflect a different composition. As a matter of fact, MLO proteins are considered negative regulators of vesicle‐associated and actin‐dependent defence pathways (Panstruga, 2005), which, once under the control of the fungus, induce actin filaments to supply nutrients for the growing hyphae (Miklis *et al*., 2007). The hypothesis is that in apple wild types, after penetration the pathogen controls the transport of material to the cell wall, changing the composition of the papillae and turning them into noneffective.

To further understand the effect of the knock‐down of *MLO* genes in apple, the expression of 17 genes involved in defence and interaction with other apple pathogens, such as *Erwinia amylovora* (fire blight), was analysed. Five genes involved in a variety of processes were down‐regulated in TG11+19 in the absence of PM infection. Among them, *MdAPOX* (*ascorbate peroxidase*), *MdGST* (*glutathione S‐transferase*) and *MdLOX* (*lipoxygenase*) have a role in plant immunity, suggesting a moderate and unexpected inhibition of defence against PM. Other down‐regulated genes were *MdALS2* and *MdNPF3.2*. Interesting was the case of *MdNPF3.2,* the homologue of a grapevine nitrite/nitrate transporter that in grapevine is up‐regulated upon PM inoculation. It has been suggested that the up‐regulation of this gene is due to the PM pathogen that, lacking in nitrate transporters and nitrite and nitrate reductases, uses those of the host to obtain ammonium, amino acids and peptides (Pike *et al*., 2014). This seems not the case of apple, as neither *MdNPF3.1* nor *MdNPF3.2* were up‐regulated in ‘Gala’ upon the inoculation with *P. leucotricha*. These results suggest that the knock‐down of *MLO* genes affected the expression of other disease‐related genes in the absence of PM infection. It was no surprise that more genes were down‐regulated in TG11+19 rather than in TG19, because of the double knock‐down in the former. However, down‐regulation in TG11+19 of three genes involved in plant defence against pathogen was unexpected: *MLO* genes are, in fact, negative regulators of defence, and the expectation was that their knock‐down would cause an activation of defence.

Considering again the expression of the 17 genes related to plant defence, a rationale is difficult to highlight based on the analysis of the three lines together. Nevertheless, the analysis of the PM inoculation effects on each of the three lines considered independently clarifies some details: line TG11+19 is extremely responsive to PM with an up‐regulation of 13 genes of 17 at 24 hpi. Two of these genes are pathogenesis related (*MdPR1* and *MdPR2)* and seven are involved in defence (*MdATPase, MdAPOX, MdLOX, MdWRKY30, MdGST, MdVSP1* and *MdVSP2*). Conversely, TG19 showed a limited transcriptional response, possibly due to its better capacity to control PM infection. The absence of gene up‐regulation at 10 dpi of most of the genes tested indicated that the transcriptional response, when evident, is more intense in early stages of pathogenesis.

In this study, results are presented concerning 18 phenolic secondary metabolites, mostly flavonoids, identified and quantified as in Vrhovsek *et al*. (2012) in the leaves of ‘Gala’, TG11+19 and TG19. For chlorogenic acid, rutin, kaempferol‐3‐*O*‐rutinoside and isorhamnetin‐3‐*O*‐glucoside, significant differences between ‘Gala’ and *mlo*‐resistant lines were found. Chlorogenic acid is known to increase potato resistance to *Streptomyces scabies*,* Verticillium albo‐atrum* and *Phytophthora infestans* (Lattanzio *et al*., 2006); it was present in lower amounts in TG11+19 compared with ‘Gala’. Kaempferol inhibits spore germination of the rice pathogen *Pyricularia oryzae* (Padmavati *et al*., 1997): an accumulation of kaempferol‐3‐*O*‐rutinoside was detected in TG19. A putative defence‐related role of rutin (present in lower amount in TG19) and isorhamnetin (accumulating in both *mlo* lines) is not at the moment known. The higher amount of isorhamnetin derivatives in TG19 may indicate an increased activity of the 3′‐methyl transferase that catalyses the methylation of quercetin to isorhamnetin. It is unlikely that the different accumulation of phenolic metabolites in the resistant lines was directly caused by the knock‐down of *MdMLO11* and *MdMLO19*. More likely, it was a secondary effect connected to the improved resistance to *P. leucotricha*.

Our results showed that *MdMLO19* is the S‐gene for PM in apple and its knock‐down substantially reduced PM susceptibility of *M. domestica*. The knock‐down of *MdMLO11,* alone or in combination with *MdMLO19,* did not cause a reduction or an additional reduction of susceptibility compared with *MdMLO19* alone; therefore, the gene did not contribute to PM resistance. Immunity to PM was not observed, as expected because of the incomplete silencing of *MLO* genes in RNAi‐transformed plants. At the level of MLO knock‐down reported, no altered pleiotropic phenotypes were detected in *mlo* plants under the adopted glasshouse conditions.

This work provides crucial information that can be used to introduce durable resistance to *P. leucotricha* in apple. This can be carried out via genome editing of *MdMLO19*, resulting in knockout mutants resistant to PM, or via the search in *M. domestica* and in wild *Malus* species of nonfunctional *MdMLO19* alleles.

## Experimental procedures

### *MdMLO18* complementation test of *A. thaliana mlo* mutant

A full‐length *MdMLO18* gene was amplified from an apple (cultivar Gala, susceptible to PM) cDNA library using the primer pair: Fw 5′‐ATGGCTGGAGACAACGGAGCTGCAA‐3′ and Rv 5′‐GAACCATTATTTTGCTGTACCTCAGCTGCC‐3′. The gene was cloned into gateway pENTR/SD‐TOPO (Thermo Fisher Scientific, Waltham, MA) and pK2WG7 vector (Life Technologies, Waltham, MA). Final constructs were verified by sequencing and inserted into *Agrobacterium tumefaciens* strain AGL0 through electroporation. *A. tumefaciens*‐transformed cells were tested by PCR for the presence of the constructs, using primers annealing on the vector and on the *MdMLO18* sequence.

The *A. thaliana Atmlo2/6/12* mutant in Col‐0 genetic background (Consonni *et al*., 2006) was grown at 25 °C in chambers with 16‐h light/8‐h dark cycle.

Gene transfer to *A. thaliana* was carried out twice (GT‐A and GT‐B) with the floral dip method (Clough and Bent, 1998), and transformed seeds were selected on kanamycin. Expression of *MdMLO18* was assessed by qPCR on leaves collected from regenerated plants. Two regenerated lines (lines A and B) were selected, one for each gene transfer.

The disease severity assessment of *A. thaliana* plants followed their inoculation by dry‐brushing leaves with *Oidium neolycopersici* spores carried by diseased tomato leaves. Disease severity was visually evaluated on all leaves 7 days postinoculation (dpi), and expressed for each plant as the mean percentage (intervals of 5%) of adaxial leaf area covered by PM mycelium.

### Constructs for *MdMLO11* and *MdMLO19* knock‐down in apple

Gene fragments for RNAi were amplified from *MdMLO11* and *MdMLO19* (accession numbers in Table S3) with primers listed in Table S4 and cloned in gateway pENTR/SD‐TOPO (Thermo Fisher Scientific). In addition, a chimeric construct was developed joining RNAi fragments supposed to silence *MdMLO11* and *MdMLO19* simultaneously (Abbott *et al*., 2002). For this purpose, a restriction site for EcoR1 was added at the 3′ end of the *MdMLO11* RNAi fragment and at the 5′ end of the *MdMLO19* one. Both fragments were restricted to EcoR1 and joined with a T4 DNA ligase (New England Biolabs, Ipswich, MA). The resulting construct was cloned into the pENTR vector. After sequencing, all fragments were cloned into the destination vector pHELLSGATE12. The fragments were inserted twice in opposite directions in each plasmid, with an intron separating them, a structure called ‘inverted repeats’ (Figure S10). The intron allows the formation of a hairpin structure that is meant to increase the knock‐down efficiency (Thermo Fisher Scientific). The final constructs were verified by sequencing and inserted into *A. tumefaciens* strain AGL0 through electroporation. *A. tumefaciens*‐transformed cells were tested by PCR for the presence of the constructs, using specific primers designed to anneal on vector and *MLO* sequences.

### Development of RNAi apple plantlets

The RNAi constructs were transferred into apple as described by Joshi *et al*. (2011). Explants from the top four leaves of 4‐week‐old *in vitro* propagated shoots of the cultivar Gala were kept on a medium with kanamycin (Joshi *et al*., 2011) and grown in a growth chamber with 16‐h light/8‐h dark cycle at 24 °C. To certify the presence of the constructs with PCR, genomic DNA from regenerated plantlets was extracted with the Illustra Nucleon Phytopure Kit (GE Healthcare). The forward primer annealed on the CaMV 35S promoter (5′‐CGCACAATCCCACTATCCTT‐3′), and the reverse primers were specific for the RNAi fragments (Table S4). PCR was performed with GoTaq^®^ Green Master Mix (Promega, Fitchburg, MA). Plants positive for the construct were moved to shoot propagation medium (SPM): 4.4 g/L of Murashige and Skoog medium with vitamins, 30 g/L of sucrose, 0.7 mg/L of 6‐benzylaminopurine (BAP), 96 mg/L of FeEDDHA, 0.8% agar, pH 5.8. New emerging shoots were propagated in fresh SPM. A total of 10–15 shoots for each line were selected for the rooting and acclimation process. To promote rooting, plants were transferred on a medium containing indole‐3‐butyric acid (IBA): 2.2 g/L of Murashige and Skoog medium with vitamins, 15 g/L of sucrose, 0.05 g/L myo‐inositol, 0.1 mg/L IBA, 0.8% agar, pH 5.8. Roots were formed approximately in 1 month from the transfer to the rooting medium, and plants were progressively acclimated to glasshouse conditions (25 °C, 16‐h light/8‐h dark cycle, relative humidity 70% ± 5%) in 125‐mL pots covered with plastic bags and containing wet autoclaved turf (‘Terriccio Vegetal Radic’—Tercomposti Spa, Brescia, Italy). Every 5–7 days for 3 weeks, air humidity was reduced to promote the formation of the foliar cuticle. Plastic bags were then removed, and plants were transferred to 1‐L pots. The control (untransformed *in vitro* grown ‘Gala’) was acclimated as described above.

### *P. leucotricha* inoculation and disease severity assessment in apple

To produce a PM inoculum, local strains of *Podosphaera leucotricha* were isolated from infected leaves of an orchard located in Trento Province (Italy). The fungus was maintained by serial inoculations on *M. domestica* seedlings under glasshouse conditions. Plants were dry‐inoculated by brushing the adaxial epidermis with leaves of infected seedlings. This system was chosen aiming at a successful infection as spores immersed in water are not always able to germinate (Turechek *et al*., 2004). To promote the fungal penetration, plants were incubated in a glasshouse at 25 °C with a relative humidity of 90% ± 5% for 6 h. The plants were then maintained at 25 °C and 80% ± 10% relative humidity until the end of the evaluation.

Four inoculation experiments were carried out in different periods of the year. In winter, the progression of infection was slightly delayed, with no differences between lines to this regard. In each test, three to eight biological replicates of each transgenic line were considered. Lines were tested in at least three of four experiments and the total number of replicates varied between 15 and 24 (Table 1). Disease severity was visually assessed on all inoculated leaves 7, 14 and 21 dpi. Disease severity was expressed as the percentage (intervals of 5%) of adaxial leaf area covered by the PM mycelium, and a single plant mean value was calculated. Reduction of disease severity in transformed plants was expressed as [(severity in controls−severity in transgenics)/severity in controls] × 100%. To consider all time points together, the area under disease progress curve (AUDPC) was calculated. AUDPC is a quantitative summary of disease intensity over time and it is the area underlying the curve representing the progress of disease severity over time (Campbell and Madden, 1990; Madden *et al*., 2007).

The number of *P. leucotricha* conidia present on infected leaves was assessed as in Angeli *et al*. (2012) with slight modifications: three leaves were collected from each replicate at 21 dpi, and four discs of 0.8 cm diameter for each leaf were cut for a total of 12 per replicate. Leaf discs were transferred to 50‐mL tubes containing 5 mL distilled water with 0.01% Tween‐20 (Sigma‐Aldrich, St. Louis, MO). Tubes were vortexed for 1 min, and the concentration of conidia per mL was determined by counting with a hemocytometer under a light microscope (Wetzlar H 600LL, Wetzlar, Germany). The amount of conidia was expressed as number per square centimetre (cm^2^) of leaf.

### Histological analysis of inoculated apple leaves

Two inoculated leaves for each replicate were collected at 3, 10 and 21 days postinoculation for bright‐field microscopy observations. To visualize fungal hyphae, leaves were cleared in ethanol–acetic acid (3:1 v/v) until chlorophyll removal (approximately 48 h). Samples were stained for 15 min with 250 μg/mL trypan blue in lactic acid, glycerol and water (1:1:1). After rinsing and mounting as in Vogel and Somerville (2000), hyphae were visualized under bright‐field illumination of a Leica LMD7000 microscope (Wetzlar, Germany).

Leaves considered for scanning electron microscopy (Hitachi S‐2300, Tokyo, Japan) were fixed in Sorensen phosphate buffer 0.1 m, pH 7, 3% glutaraldehyde. After 24 h, leaves were washed in Sorensen buffer without glutaraldehyde for 2 h under mild agitation (80–100 rpm). Afterwards, samples were progressively dehydrated with four ethanol washings at concentrations from 40% to 100%, dried and kept in Falcon tubes until observation. Fragments of leaves were metallized with gold before observation. Images were processed with ImageJ software (http://imagej.nih.gov/ij/).

For the detection of papillae, leaves were cleared in ethanol–acetic acid (3:1, v/v) until chlorophyll removal and equilibrated overnight in a solution of lactic acid, glycerol and water (1:1:1). Papillae were visualized using the LMD filter (BP filter 380‐ to 420‐nm excitation, 415 dichroic mirror and BP 445‐ to 485‐nm emission) of a Leica LMD6500 microscope (Leica Microsystem, Wetzlar, Germany).

### Gene expression analysis

To identify lines showing silencing effects, a first gene expression study used triplicates of *in vitro* grown transgenic plants. In the second study, concerning acclimated transgenic plants, leaf samples were collected immediately before PM inoculation, at 24 hpi and at 10 dpi. For each line at each time point, leaf samples were collected from five different plants. Samples were frozen in liquid nitrogen and stored at 80 °C. Total RNA was extracted with the Spectrum™ Plant Total RNA Kit (Sigma‐Aldrich), treated with the DNAse I (Sigma‐Aldrich) and reverse‐transcribed using the SuperScript III reverse transcriptase (Invitrogen, Life Technologies, Waltham, MA). The qPCR analyses were run according to SsoAdvanced Universal SYBR Green Supermix (Bio‐Rad, Hercules, CA) in a 15‐μL reaction volume, using a CFX96 Touch™ Real‐Time PCR Detection System (Bio‐Rad) and the CFX Manager software. Samples were run in two technical replicates according to the following thermal cycling parameters: 95 °C 3 min, 95 °C 10 s, 55 °C 30 s (repeated 40 times), 95 °C 10 s. For the analysis of *MdMLO19,* the primer pairs considered in previous work were used (Table S3; Pessina *et al*., 2014). For *MdMLO11* and for the expression of 17 genes involved in the interaction between apple and *P. leucotricha*, new primer pairs were designed with the NCBI Primer Designing Tool (http://www.ncbi.nlm.nih.gov/tools/primer-blast/) (Table S3). Serial dilutions of cDNA (1/10, 1/100, 1/1000 and 1/10 000) allowed to calculate the efficiency of the primer pairs; the expected sizes of the products were confirmed using agarose gel electrophoresis. Presence of a specific final dissociation curve was determined after every qPCR run, with progressive increments of temperature from 65 °C to 95 °C (0.5 °C each step, 5 s). The reference genes considered were elongation factor 1, ubiquitin and *8283* (Table S3). All of them are known to be stable reference genes for apple (Botton *et al*., 2011; Pessina *et al*., 2014). The analysis with the software geNorm (medgen.ugent.be/~jvdesomp/genorm) resulted in M‐values lower than 1 for all three reference genes, in conditions where M‐values lower than 1.5 are considered adequate (Ling and Salvaterra, 2011). The threshold cycles (Ct) were converted to relative expression levels as in Hellemans *et al*. (2007), using as input the average Ct of the two technical replicates. As reference Ct, the average Ct of wild‐type ‘Gala’ at 0 hpi was adopted.

### Phenolic metabolites

Quantification of phenolic metabolites in transgenic and wild‐type apple plants was carried out on noninoculated leaves from eight biological replicates. Samples were ground in liquid nitrogen, and 100 mg of powder was used for the extraction in 4 mL of 100% methanol of the target metabolites. Extraction lasted 72 h at 4 °C. The liquid phase was diluted with water to 80% methanol and filtered with 13‐mm Millex‐GV syringe filters (Millipore, Billerica, MA) to remove fine debris. The quantification of 18 phenolic metabolites was carried out by multiple reactions monitoring (MRM) as described by Vrhovsek *et al*. (2012).

### Statistics

#### Disease severity

Severity data were analysed by the statistical package SPSS (IBM, Armonk, NY). For both apple and *A. thaliana,* severity data of leaves from the same plant were averaged before further analyses. Apple severity data of the eight younger leaves of a plant were considered, while *A. thaliana* data were from all leaves. Before any analysis, data were shown to be normally distributed (Kolmogorov–Smirnov and Shapiro–Wilk tests *P* > 0.05) and to have homogeneous variances (Levene's test, *P* > 0.05). One‐way ANOVA with Tukey's *post hoc* test was adopted to detect significant differences (*P* < 0.05) at each time point. Data were transformed according to *y* = arcsin(*x*), in order to meet the prerequisites of ANOVA. In case of nonhomogeneous variances, the Games–Howell *post hoc* test was applied. Prior to pooling data from independent experiments, the effect of single experiments was tested: no significant effect of the experiments emerged. Pooled data were analysed independently for time points 14 and 21 dpi. AUDPC data were treated as described above for severity data. Number of conidia data was analysed with one‐way ANOVA, applying the Tukey *post hoc* test (*P* < 0.05).

#### qPCR data analyses

For the evaluation of gene expression, relative expression values were transformed in logarithmic scale according to *y* = ln(*x*) (Pessina *et al*., 2014) to meet normal distributions and homogeneities of variances, as assessed, respectively, with the test of Shapiro–Wilk (*P* ≤ 0.05) and Levene (*P* ≤ 0.05). Pairwise comparison of homoscedastic data was carried out with Tukey's test (*P* < 0.05), whereas nonhomoscedastic data were analysed with the Games–Howell test (*P* < 0.05), using the statistical package SPSS (IBM). Defence gene expression analysis was tested with the Fisher *post hoc* test.

#### Correlations

The two‐tailed Pearson's correlation test was adopted to investigate the correlations between AUDPC and relative expression of *MLO* genes at 10 dpi, and between degree of severity and number of conidia, both at 21 dpi. All data have been transformed following *y* = arcsin(*x*) to achieve a normal distribution.

#### Metabolites

The data from the phenolic metabolites were subjected to one‐way ANOVA with the Fisher *post hoc* test. In case of nonhomoscedastic data, the Games–Howell *post hoc* test was applied and the Kruskal–Wallis nonparametric test for data not normally distributed.

## Author contribution

SP carried out the *A. thaliana* complementation test, built the constructs for apple gene transfer, developed the transgenic plants, performed the gene expression analyses, the microscopy characterization, the conidia count and the data analyses and wrote the major part of the manuscript. DA carried out the severity assessment in the glasshouse, contributed to the data analysis and reviewed the manuscript. SM identified and quantified phenolic metabolites and reviewed the manuscript. FS inspired the work here presented and revised the manuscript. YB contributed to the planning of the project and to the design of some experiments and revised the manuscript. RGFV contributed to the design of the experiments and revised the manuscript. RV contributed to the design of the experiments and revised the manuscript. HJS contributed to the design of the experiments and to data analysis and revised the manuscript. MM contributed to the design of the experiments, contributed to data analysis and was the main reviewer of the manuscript.

## Authors' disclosure of potential conflict of interest

The authors declare that on 8 July 2015 a patent protecting *MdMLO* genes has been submitted to the European Patent Office (EPO). The patent application number is PCT/EP2015/073135.

## Supporting information


**Figure S1.** Arabidopsis plants inoculated with O. *neolycopersici*.

**Figure S2.** Expression of *MdMLO11* and *19* in 41 *in vitro* transgenic lines.

**Figure S3.** Expression of *MdMLO11* and *19* in 12 transgenic lines acclimated to greenhouse conditions.

**Figure S4.** Infection severity of four apple *mlo* lines inoculated with *P. leucotricha*

**Figure S5.** Conidia count.

**Figure S6.** SEM microscopy images of infected leaves of ‘Gala’, TG0 and TG11+19.

**Figure S7.** Expression of *MdMLO5* and *7* in four *mlo* lines and control ‘Gala’ in absence of *P. leucotricha* infection.

**Figure S8.** Relative expression of 17 genes related to plant disease resistance.

**Figure S9.** Phenolic metabolites content in leaves of ‘Gala’ and resistant lines TG11+19 and TG19.

**Figure S10.** pHELLSGATE12 construct inserted in apple genome by *A. tumefaciens*‐mediated gene transfer.

**Table S1.** Summary of gene transfer results.
**Table S2.** Identified and quantified phenolic metabolites.
**Table S3.** Primers for qPCR. **Table S4.** Primers for RNAi.
