# Supplementary figures and images for: The knock‐down of the expression of MdMLO19 reduces susceptibility to powdery mildew (Podosphaera leucotricha) in apple (Malus domestica)
Source: Plant Biotechnol J. 2016 May 11;14(10):2033–44. doi: 10.1111/pbi.12562 (PMC5043462; doi:10.1111/pbi.12562)

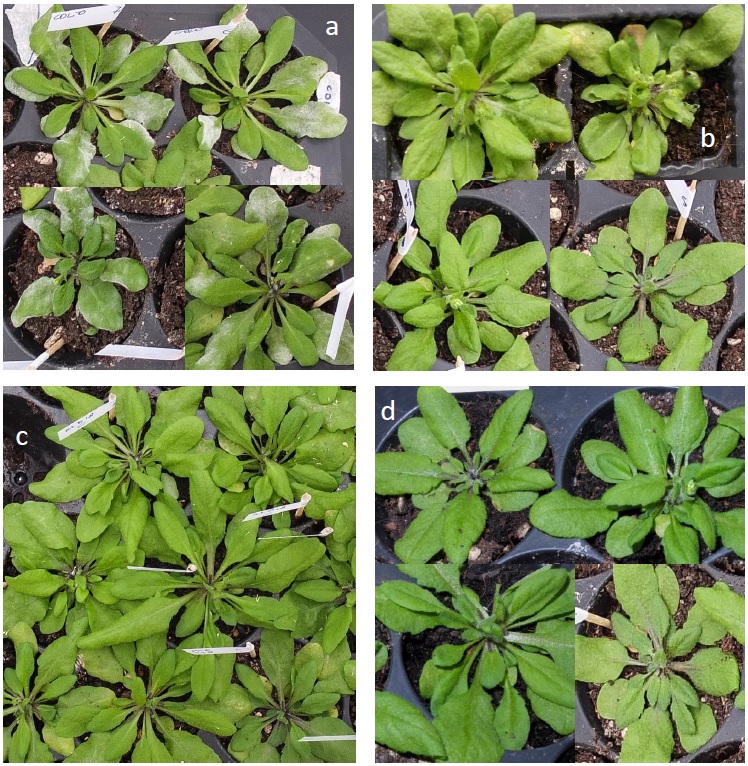

Supplement: Supplementary file 1 — Figure S1. Arabidopsis plants inoculated with O. neolycopersici. [file PBI-14-2033-s005.jpg]

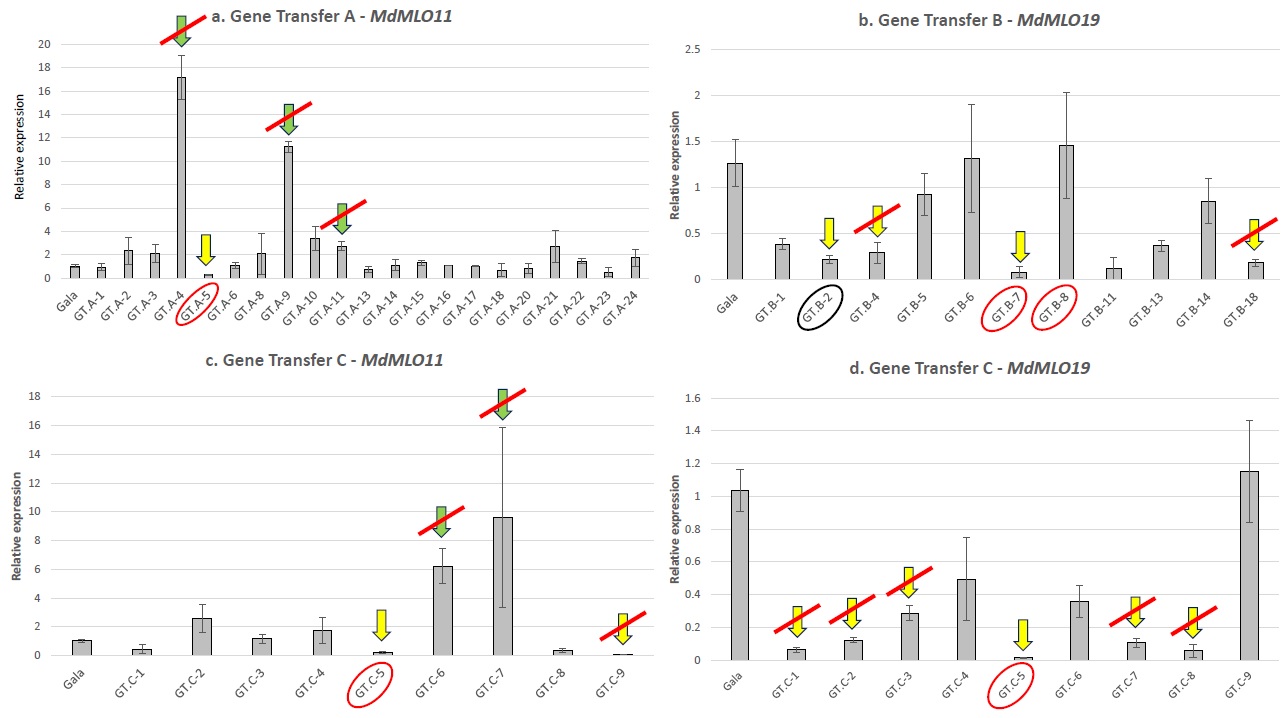

Supplement: Supplementary file 2 — Figure S2. Expression of MdMLO11 and 19 in 41 in vitro transgenic lines. [file PBI-14-2033-s009.jpg]

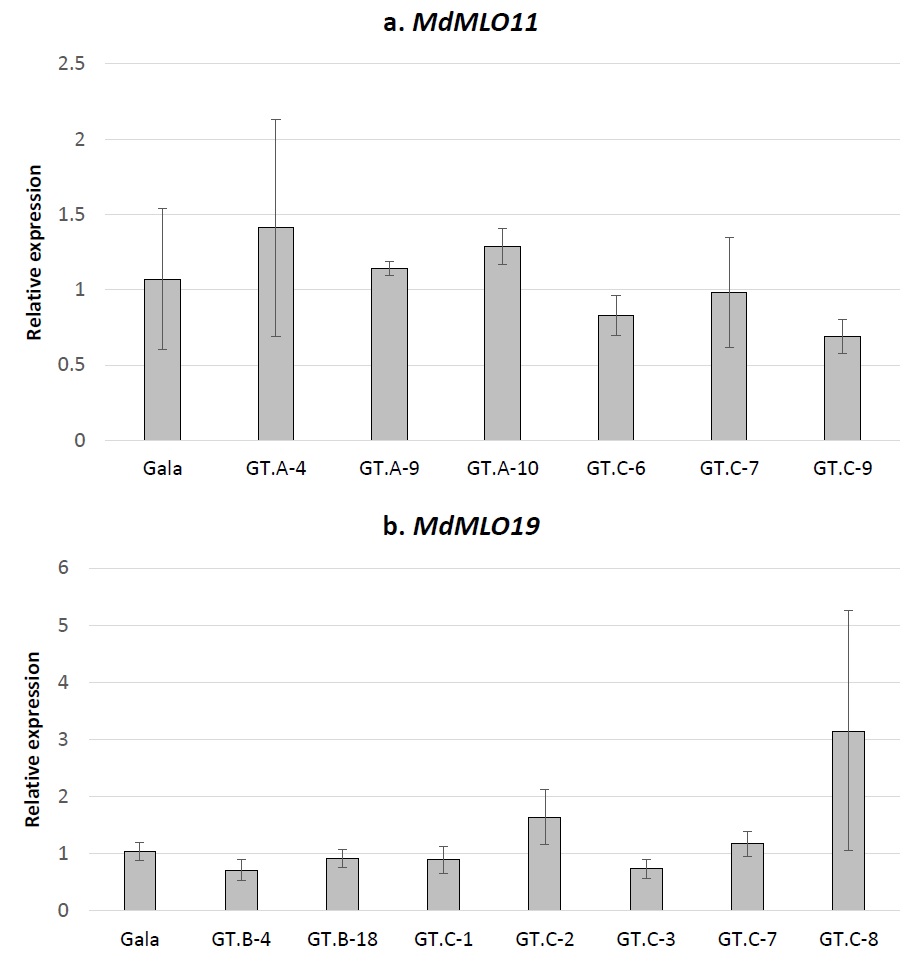

Supplement: Supplementary file 3 — Figure S3. Expression of MdMLO11 and 19 in 12 transgenic lines acclimated to greenhouse conditions. [file PBI-14-2033-s008.jpg]

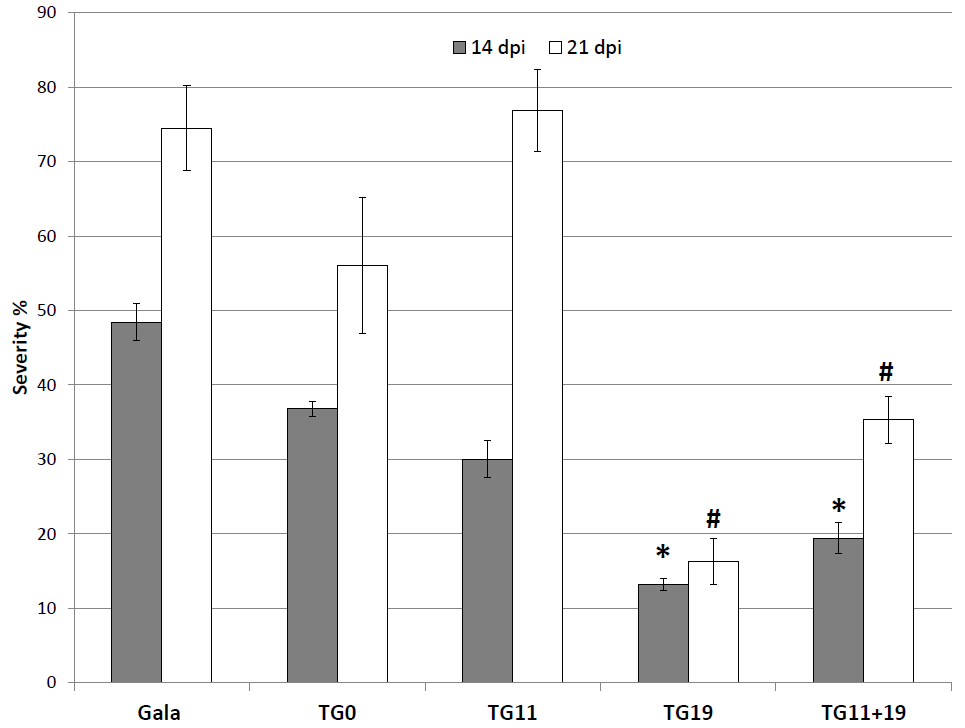

Supplement: Supplementary file 4 — Figure S4. Infection severity of four apple mlo lines inoculated with P. leucotricha [file PBI-14-2033-s010.tif]

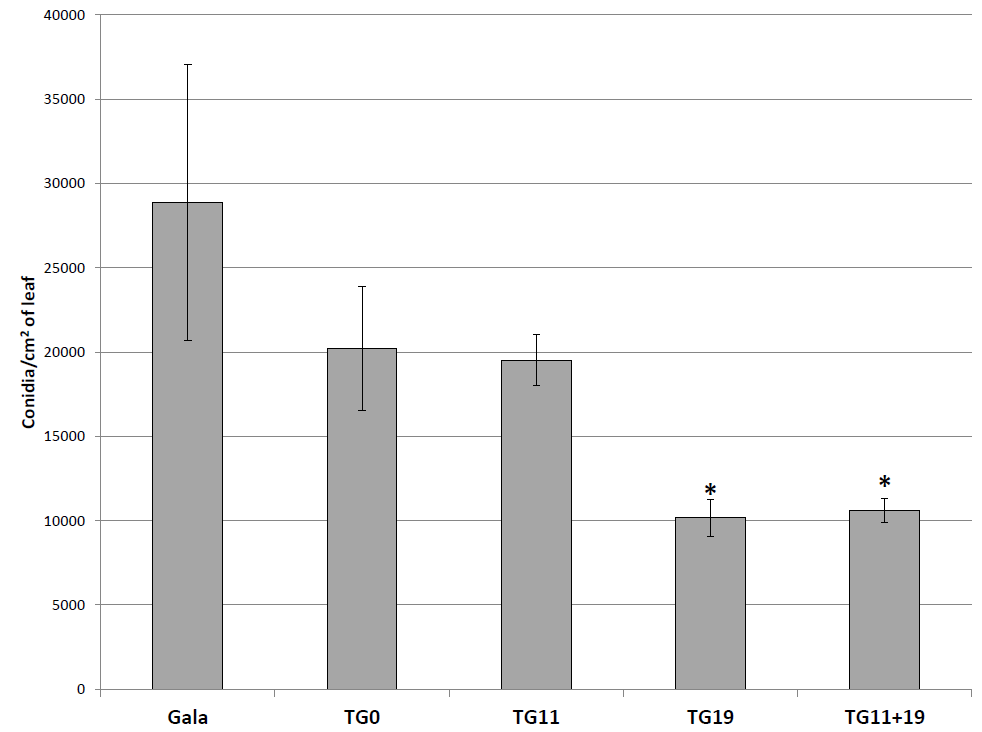

Supplement: Supplementary file 5 — Figure S5. Conidia count. [file PBI-14-2033-s001.tif]

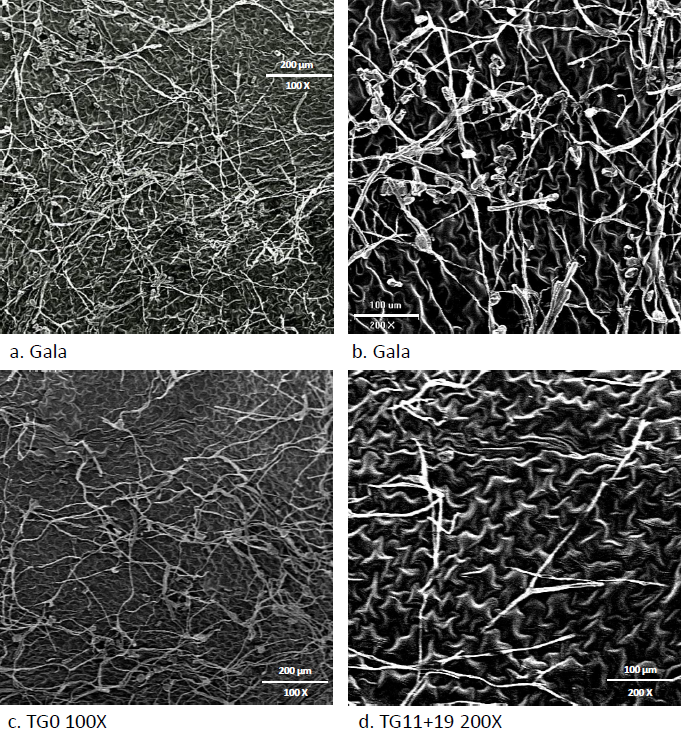

Supplement: Supplementary file 6 — Figure S6. SEM microscopy images of infected leaves of ‘Gala’, TG0 and TG11+19. [file PBI-14-2033-s002.tif]

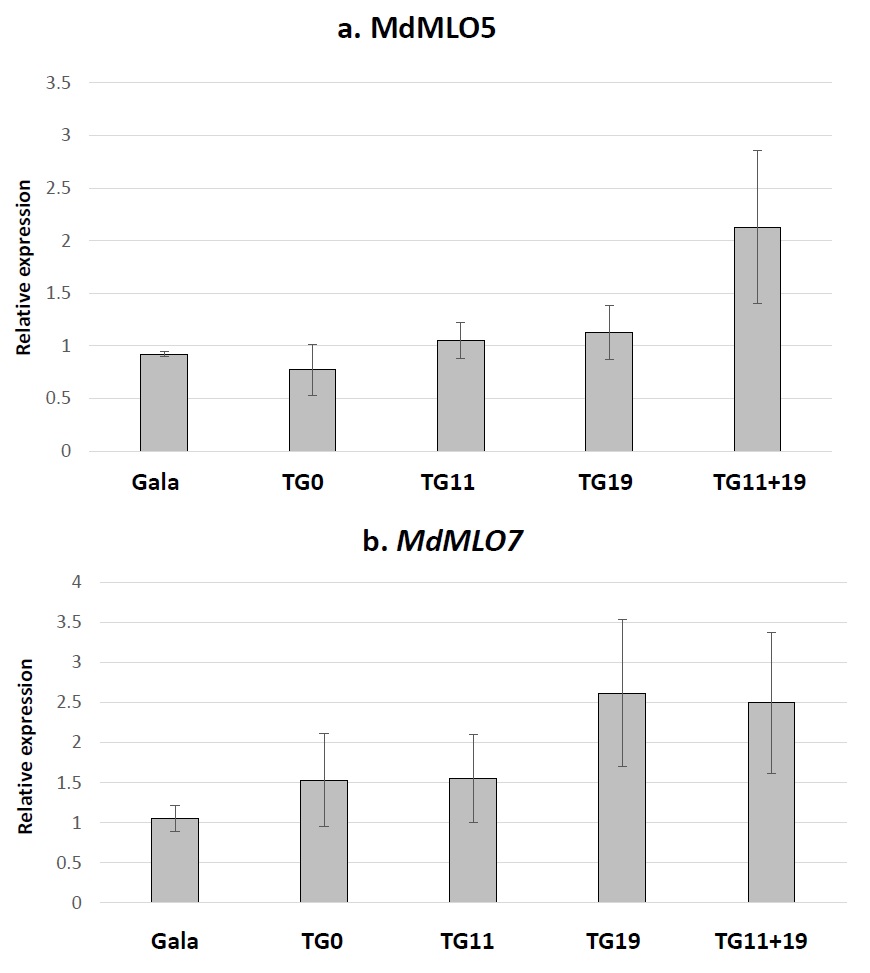

Supplement: Supplementary file 7 — Figure S7. Expression of MdMLO5 and 7 in four mlo lines and control ‘Gala’ in absence of P. leucotricha infection. [file PBI-14-2033-s003.jpg]

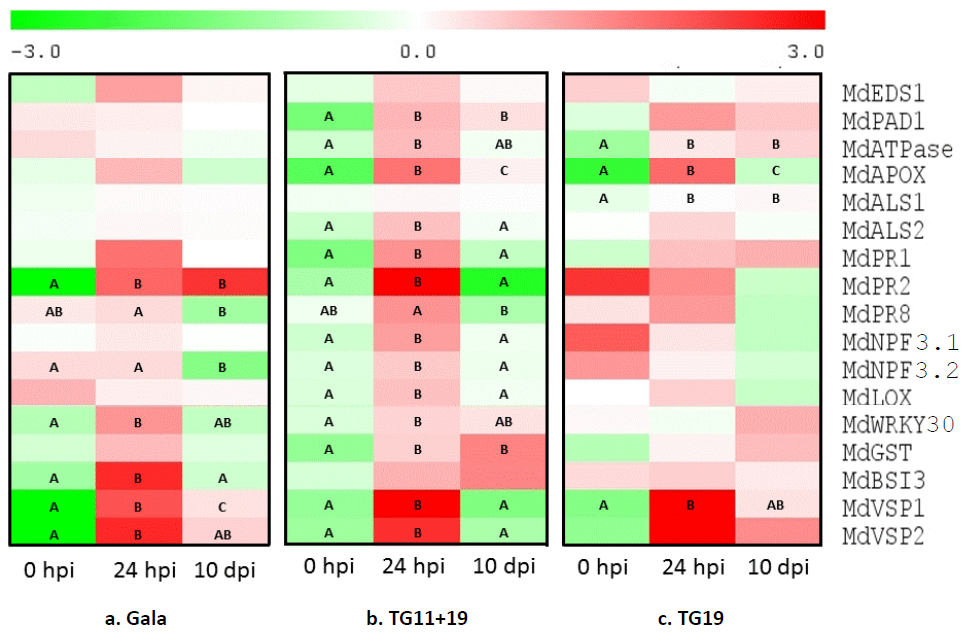

Supplement: Supplementary file 8 — Figure S8. Relative expression of 17 genes related to plant disease resistance. [file PBI-14-2033-s004.tif]

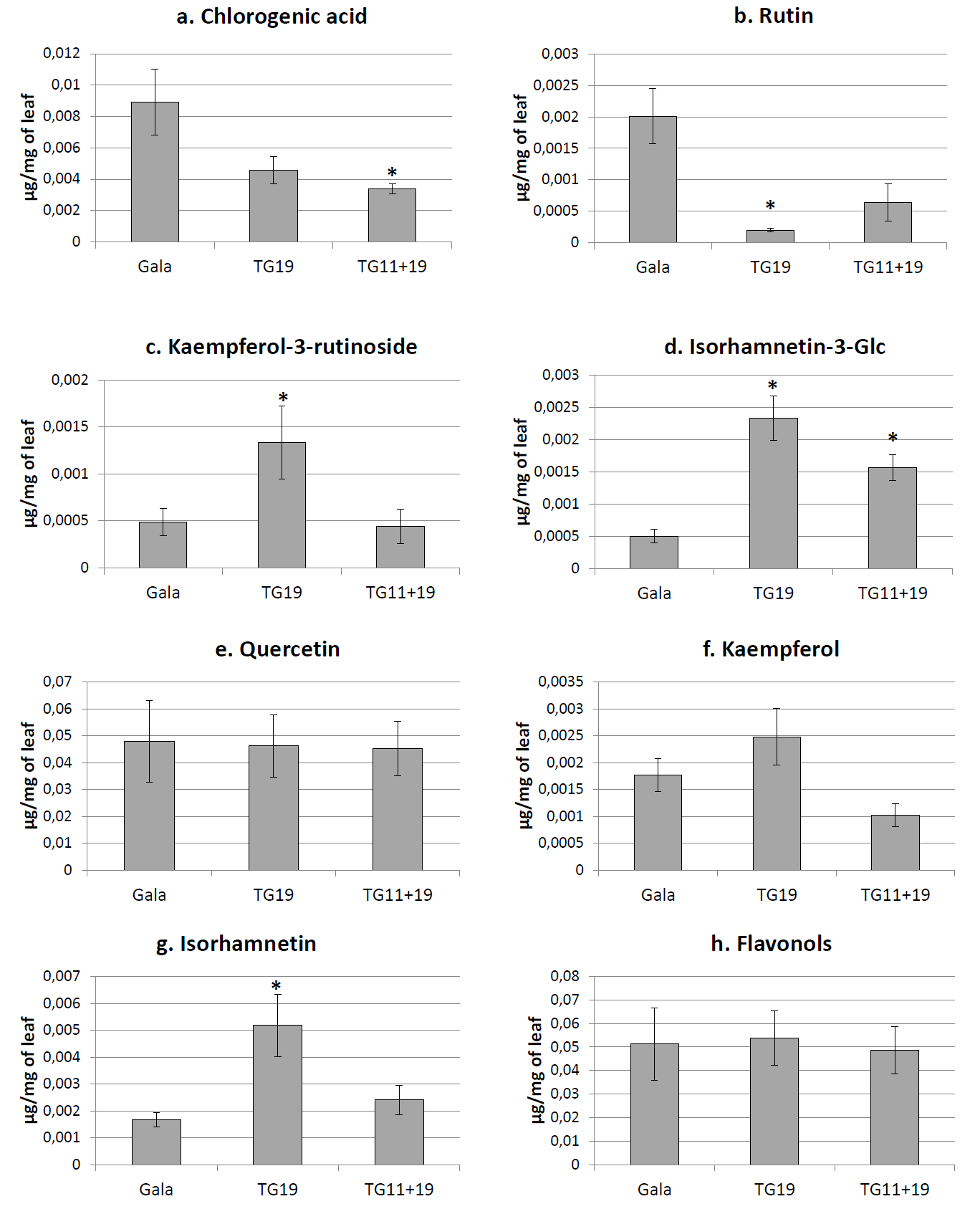

Supplement: Supplementary file 9 — Figure S9. Phenolic metabolites content in leaves of ‘Gala’ and resistant lines TG11+19 and TG19. [file PBI-14-2033-s011.tif]

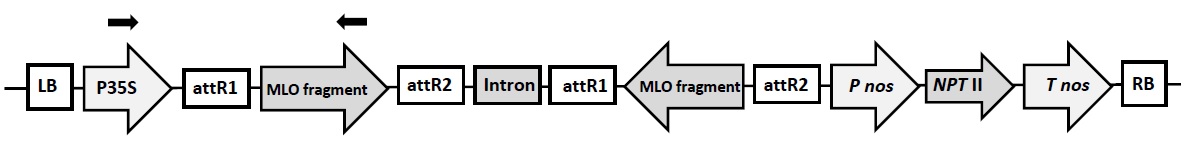

Supplement: Supplementary file 10 — Figure S10. pHELLSGATE12 construct inserted in apple genome by A. tumefaciens‐mediated gene transfer. [file PBI-14-2033-s006.jpg]
