# Supplementary material for: The knock‐down of the expression of MdMLO19 reduces susceptibility to powdery mildew (Podosphaera leucotricha) in apple (Malus domestica)
Source: Plant Biotechnol J. 2016 May 11;14(10):2033–44. doi: 10.1111/pbi.12562 (PMC5043462; doi:10.1111/pbi.12562)
Supplement: Supplementary file 11 — Table S1. Summary of gene transfer results. Table S2. Identified and quantified phenolic metabolites. Table S3. Primers for qPCR. Table S4. Primers for RNAi. [file PBI-14-2033-s007.docx]

**SUPPORTING INFORMATIONS**

| **Table S1**: summary of gene transfer results | | | | |
| --- | --- | --- | --- | --- |
| **Gene transfer** | **Knocked-down gene** | **Regenerated lines** | **Confirmed transgenic** | **Selected**  **lines** |
| A | *MdMLO11* | 39 | 23 | TG11 |
| B | *MdMLO19* | 33 | 19 | TG0, TG19 |
| C | *MdMLO11* + *MdMLO19* | 8 | 5 | TG11+19 |

| **Table S2** identified and quantified phenolic metabolites | |
| --- | --- |
| **Compound** | **Group (subgroup)** |
| Chlorogenic acid | Benzoic acids |
| Coniferyl alcohol | Phenylpropanoids |
| Phloretin | Polyketides (Dihydrochalcones) |
| Phlorizin | Polyketides (Dihydrochalcones) |
| Naringenin | Flavonoids (Flavanones) |
| Catechin | Flavonoids (Flavan-3-ols) |
| Epicatechin | Flavonoids (Flavan-3-ols) |
| Procyanidin B2 + B4 | Flavonoids (Proanthocyanidins) |
| Kaempferol | Flavonoids (Flavonols) |
| Kaempferol-3-*O*-glucoside | Flavonoids (Flavonols) |
| kaempferol-3-*O*-rutinoside | Flavonoids (Flavonols) |
| Isorhamnetin | Flavonoids (Flavonols) |
| Isorhamnetin-3-*O*-glucoside | Flavonoids (Flavonols) |
| Isorhamnetin-3-*O*-rutinoside | Flavonoids (Flavonols) |
| Quercetin-3-*O*-rhamnosid | Flavonoids (Flavonols) |
| Quercetin-3-*O*-glucoside | Flavonoids (Flavonols) |
| Quercetin-3-*O*-galactoside | Flavonoids (Flavonols) |
| Rutin | Flavonoids (Flavonols) |
| Arbutin | Hydroquinones |

| **Table S3:** Primers for qPCR | | | | |
| --- | --- | --- | --- | --- |
| **Name** | **Accession number** | **Forward (‘5 – ‘3)** | **Reverse (‘5 – ‘3)** | **Reference** |
| *EF1* | MD09G014760**^a^** | TACTGGAACATCACAGGCTGAC | TGGACCTCTCAATCATGTTGTC | Pessina et *al.* (2014) |
| *Ubiquitin* | MD05G001920**^a^** | CATCCCCCCAGACCAGCAGA | ACCACGGAGACGAAGCACCAA | Pessina et *al.* (2014) |
| *Md8283* | MDP0000375455^b^ | CTCGTCGTCTTGTTCCCTGA | GCCTAAGGACAGGTGGTCTATG | Botton *et al.* (2011) |
| *MdMLO11* | MDP0000239643**^b^** | ATCGAAGGCTGTTGGAGCAA | AAGCACGTGAAAGACGGCTA | / |
| *MdMLO19* | MDP0000168714**^b^** | CAGAGTGGCGACTGCACTTA | GGGACATGGAGTGCAAAGGA | Pessina et *al.* (2014) |
| *MdALS1* | MDP0000621545**^b^** | TACAAGTACAAGCGGCCTCG | TCTCCACACTGATCGTTGCC | / |
| *MdALS2* | MDP0000715451**^b^** | ATGTCTCGGTTTGTGCGGAT | GGAGGAATGGCTTCCCCAAA | / |
| *MdNPF3.1* | MDP0000606453**^b^** | GCTGGCCCTAATGGATCGAA | TTTTCTCCAGACTCGGCACC | / |
| *MdNPF3.2* | MDP0000549956**^b^** | CAACGCAGCTTCATATGCCC | ATAGTCCAGAAGCGGCCAAC | / |
| *MdVSP1* | MDP0000155698**^b^** | AGCTTTGCCCGAGAGTCTTC | ACAAGCTTCTCCCAGGTGTG | / |
| *MdVSP2* | MDP0000274344**^b^** | GGCACCAGTACAGGAAGGAC | TGAATGCCTTCGTCCCGAAT | / |
| *MdLOX* | MDP0000300321^b^ | GTTGCGTATGGGAAGGAATGG | GGTAGTAGTGGTTTACATAGTCAGTG | / |
| *MdPAD1* | MDP0000158955**^b^** | TCAATGACTGGGCAATCGCA | TCCAGGGCAAACTCTTCGAC | / |
| *MdWRKY30* | MDP0000468391^b^ | AATATACATTGGGAGCAAAAGAGTC | AGAGTTCAGCATGGAAAGCG | / |
| *MdATPase* | MDP0000494888^b^ | TCTTTCTTCCACCTGCTCCT | GTTCTCACACACAATCTTCCCATCC | / |
| *MdAPOX* | MDP0000241173^b^ | GCTCCAACTGACAAGGCTCTTC | CGCACAGGCATCGGCTTC | / |
| *MdBSI3* | MDP0000287919^b^ | CGCCTTTGGTTAAGAATGAGCCTC | CCTCAATGTTACAAATGTCTTGGCGC | / |
| *MDGST* | MDP0000266097^b^ | GAGCCCTTCATATCCCTCAATCC | GCCTCCACCTCCGACCAC | / |
| *MdEDS1* | MDP0000479863 | TGGAGAAAGTGATTTTGGAGAAGC | AGAACCAGATTGTGACAAACGC | / |
| *MdPR1* | AF507974^c^ | AGCACACGAGTTCGACTCATAA | CACAAAACTACGCCAACCAA | / |
| *MdPR2* | AF494404^c^ | GGTCGGTGGAGGATCTTTGG | TTGGAGTCCCTCCCTTCACA | / |
| *MdPR8* | DQ318214^c^ | CCAAGCCCCTGTCCTAAACCTC | CAACTTGCCTTGCCTCATCAGC | / |

**^a^** Available at http://bioinformatics.psb.ugent.be/plaza

**^b^** Available at http://www.rosaceae.org/gb/gbrowse/malus_x_domestica/

^c^ Available at http://www.ncbi.nlm.nih.gov/genbank/

| **Table S4**: Primers for RNAi | | | | |
| --- | --- | --- | --- | --- |
| **Gene** | **Accession number^a^** | **Primer Forward** | **Primer Reverse** | **Amplicon Lenght** |
| *MdMLO11* | MDP0000239643 | GCACATCGCAGCGAAGAAGCAC | AGCTTTCAGTGTCCTGTTCGGATTG | 134 bp |
| *MdMLO19* | MDP0000168714 | TGCACTTGCTTTCTTTGCATGGAC | AACGACATCTTCCAACTTCTCATGG | 115 bp |

**^a^** Available at http://www.rosaceae.org/gb/gbrowse/malus_x_domestica/

**Figure S1.** *A. thaliana* plants after 7 days from the inoculation with *O. neolycopersici*. a) Col-0 plants are susceptible to PM. b) *Atmlo2/6/12* plants are resistant to PM. c) *Atmlo2/6/12* plant expressing *MdMLO18* resulting from gene transfer A are resistant to PM. d) *Atmlo2/6/12* plant expressing *MdMLO18* resulting from gene transfer B are resistant to PM.

**Figure S2.** Expression of *MdMLO11* and *MdMLO19* in 41 *in vitro* transgenic lines coming from three gene transfer: gene transfer A (a), gene transfer B (b) and gene transfer C (c and d). Each bar represents the line average relative expression, evaluated from two or three *in vitro* plants. Error bars show the standard errors of the mean. Significant differences in comparison with ‘Gala’ according to Tukey or Games-Howell post-hoc tests (P=0.05) are indicated by arrows: yellow arrows indicate down-regulation, green arrows up-regulation. The red bars on the arrows indicates the lines for which up and down-regulation was not confirmed after acclimation to greenhouse conditions. The black circle indicates line GT.B-2 (Gene Transfer B), which was not able to survive acclimation to greenhouse conditions. The red circles indicate the lines that were used in this study. GT.A-5 was renamed TG11, GT.B-7 became TG19, GT.B-8 became TG0 and TG.C-5 was renamed TG11+19. The red ‘x’ above the arrows indicates the lines that were lost due to in vitro contamination before it was possible to acclimate them to greenhouse conditions.

**Figure S3.** Expression of *MdMLO11* (a) and *MdMLO19* (b) in six and seven transgenic lines, respectively. Each bar represents the line average relative expression, evaluated from three to five plants. Error bars show the standard errors of the mean. No significant differences in comparison with ‘Gala’ were detected by Tukey or Games-Howell post-hoc tests (P=0.05).

**Figure S4.** Infection severity at 14 and 21 dpi of four apple *mlo* lines inoculated with *P. leucotricha*. Each bar represents the average severity of infection at the given time point, calculated on 15-23 biological replicates and four experiments. Error bars show standard errors of the mean. Dark grey bars represent severity at 14 dpi, white bars at 21 dpi. Each time point has been analysed independently. For each time point, symbols highlight significant differences compared to the control Gala, according to Tukey or Games-Howell post-hoc test (P = 0.05): * for 14 dpi, # for 21 dpi.

**Figure S5.** Number of conidia per cm^2^ leaf surface of ‘Gala’ and *mlo* lines TG0, TG11, TG19 and TG11+19 inoculated with *P. leucotricha* at 21 dpi. Bars indicate the average number of conidia, measured in two experiments. Error bars show standard errors of the mean. Asterisks indicate statistically significant differences compared to ‘Gala’ according to Tukey post-hoc test (P = 0.01).

**Figure S6:** SEM microscopy images of infected leaves of ‘Gala’ (a, b), the susceptible line TG0 (c) and the resistant line TG11+19 (d). Pictures were taken at 21 dpi.

**Figure S7.** Expression of *MdMLO5* (a) and *MdMLO7* (b) in five *mlo* lines in absence of *P. leucotricha* infection. Each bar represents the line average relative expression, evaluated from three to five plants. Error bars show the standard errors of the mean. No significant differences in the comparison of *mlo* lines with ‘Gala’, based on Tukey or Games-Howell post-hoc tests (P=0.05) were detected.

**Figure S8** Relative expression at three time points in Gala (a) and resistant *mlo* lines TG11+19 (b) and TG19 (c) of 17 genes related to plant disease resistance. Each line point was analysed independently and the average Ct of all samples was used as reference for the statistical analysis. The letter code indicates statistically significant differences among time points according to Fisher post-hoc test (P=0.05). The image was prepared with the Multiexperiment Viewer software with the Log2 of relative expression data.

**Figure S9:** Phenolic metabolites content in leaves of ‘Gala’ and resistant lines TG11+19 and TG19. The average level of chlorogenic acid (a), rutin (b), Kaempferol-3-*O*-rutinoside (c), isorhamnetin-3-*O* glucoside (d), quercetins (e), kaempferols (f), isorhamnetins (g) and Flavonols (h) from eight samples is shown. Error bars show standard errors of the mean. Asterisks indicate significant differences (P = 0.05) according to Fisher or Games-Howell post-hoc tests or Kruskall-Wallis test.

**Figure S10.** pHELLSGATE12 construct inserted in apple genome by *A. tumefaciens*-mediated gene transfer. LB: left border; P35S: promoter; attR1 and attR2: gateway recombination sites; MLO fragment: DNA fragment amplified from apple *MLO* gene(s) (*MdMLO11, MdMLO19* or *MdMLO11+MdMLO19*) aimed at knocking-down its complementary target; Intron: allows the formation of an hairpin structure, which increases the efficiency of silencing. P *nos*: *nos* promoter for the marker gene; *NPT II*: marker gene for kanamycin resistance; T *nos*: *nos* terminator for the marker gene. The two black arrows indicate the annealing sites of the primers used to certify the presence of the insert in the regenerated plantlets. The PCR amplicons were 344 bp for TG11 (210 promoter + 132 RNAi fragment), 325 bp for TG19 (210 promoter + 115 RNAi fragment) and 457 bp for TG11+19 (210 promoter + 132 RNAi fragments for *MdMLO11* + 115 RNAi fragments for *MdMLO19*).
